# Cyclovirobuxine inhibits the progression of clear cell renal cell carcinoma by suppressing the IGFBP3-AKT/STAT3/MAPK-Snail signalling pathway

**DOI:** 10.7150/ijbs.62114

**Published:** 2021-08-13

**Authors:** Yadong Liu, Huiyan Lv, Xingyi Li, Jiannan Liu, Song Chen, Yaodong Chen, Yinshan Jin, Ruihua An, Shiliang Yu, Zhigang Wang

**Affiliations:** 1 Institute of Ultrasound Imaging, The Second Affiliated Hospital of Chongqing Medical University, Chongqing 400010, China.; 2 State Key Laboratory of Ultrasound in Medicine and Engineering, Chongqing Medical University, Chongqing 400016, China.; 3 Department of Nephrology, The First Affiliated Hospital of Harbin Medical University, No.23 You Zheng Street, Harbin 150001, Heilongjiang, China.; 4 Department of Ultrasonic Imaging, Ningbo First Hospital, The Affiliated Hospital of Ningbo University, Ningbo, China.; 5 Department of Urology, The First Affiliated Hospital of Harbin Medical University, No.23 You Zheng Street, Harbin 150001, Heilongjiang, China.; 6 Department of Ultrasonic Imaging, First Clinical Medical College, Shanxi Medical University, Taiyuan, 030001, Shanxi Province, China.

**Keywords:** Cyclovirobuxine, clear cell renal cell carcinoma (ccRCC), IGFBP3, AKT/STAT3/MAPK, Snail, EMT

## Abstract

Of all pathological types of renal cell cancer (RCC), clear cell renal cell carcinoma (ccRCC) has the highest incidence. Cyclovirobuxine (CVB), a triterpenoid alkaloid isolated from *Buxus microphylla*, exhibits antitumour activity against gastric cancer and breast cancer; however, the mechanism by which CVB inhibits ccRCC remains unclear. The aim of our study was to explore the antitumour effects of CVB on ccRCC and to elucidate its exact mechanism. Cell viability, proliferation, cell cycle distribution, apoptosis, wound healing and invasion were evaluated. Furthermore, Western blotting, immunofluorescence staining, immunohistochemical staining, and bioinformatics analyses were utilized to comprehensively probe the molecular mechanisms. The *in vivo* curative effect of CVB was explored using a 786-O xenograft model established in nude mice. CVB reduced cell viability, proliferation, angiogenesis, the epithelial-mesenchymal transition (EMT), migration and invasion. In addition, CVB induced cell cycle arrest in S phase and promoted apoptosis. The expression of the EMT-related transcription factor Snail was significantly downregulated by CVB via the inhibition of the AKT, STAT3 and MAPK pathways. We revealed that insulin-like growth factor binding protein 3 (IGFBP3) was the true therapeutic target of CVB. CVB exerted anti-ccRCC effects by blocking the IGFBP3-AKT/STAT3/MAPK-Snail pathway. Targeted inhibition of IGFBP3 with CVB treatment may become a promising therapeutic regimen for ccRCC.

## Introduction

Renal cell carcinoma (RCC) is the most frequently occurring primary cancer of the kidney, with an increasing incidence in recent years, and accounts for 2-3% of all malignancies 1. Clear cell renal cell carcinoma (ccRCC), the most common histological subtype of RCC, originates from renal tubular epithelial cells and accounts for approximately 75% of all resectable renal neoplasms 2. Approximately one-third of patients with RCC present with metastatic lesions at the time of the initial diagnosis 3. Although multimodal therapeutic options, including surgical resection, radiotherapy, chemotherapy, immunotherapy and targeted therapies, have been used to treat RCC, the therapeutic effects are unsatisfactory due to the low response rate resulting from drug resistance 4. Patients with extrarenal metastasis have an unfavourable prognosis, with a 5-year survival rate of less than 10% 1. Therefore, the discovery of new therapeutic strategies for RCC is critical and urgently needed. The epithelial-mesenchymal transition (EMT) is an extremely complicated biological process in which epithelial cells lose their epithelial features, including cell polarity and intercellular tight junctions, and acquire mesenchymal characteristics, such as enhanced motility and invasive capacities 5. Downregulation of the epithelial marker E-cadherin and upregulation of mesenchymal markers (e.g., Vimentin, Fibronectin, and N-cadherin) are the most noteworthy phenotypic changes in the EMT 6. In addition, a series of transcriptional repressors (Snail, Slug, Twist, and ZEB1) manipulate the EMT process by binding to the E-box elements located in the E-cadherin promoter 7. In recent years, numerous studies have shown that the EMT participates in the occurrence and progression of multiple types of malignancies, including ovarian cancer, glioblastoma, bladder cancer and RCC 6-8. Furthermore, the EMT is strongly associated with excessive proliferation, apoptosis resistance and stem cell properties of cancer cells and is considered the critical factor for tumour invasion and migration 6,8. Thus, the development of effective therapeutic approaches for inhibiting the EMT has become a research hotspot.

Insulin-like growth factor binding protein 3 (IGFBP3), a multifunctional secreted glycoprotein, is a member of the family of six IGF binding proteins (IGFBPs) 9. As the most predominant member of this family, IGFBP3 not only transports the vast majority of insulin-like growth factor (IGF) in the circulation and regulates IGF bioavailability but also plays an important role in various cellular processes, including survival, growth, proliferation, senescence, apoptosis, differentiation and oxidative stress 10. Moreover, several studies have indicated that IGFBP3 exerts growth-stimulatory effects on many cancers, such as glioblastoma, breast cancer, prostate cancer and colorectal cancer 11,12. Overexpression of IGFBP3 in the aforementioned tumour tissues is closely related to the acceleration of growth and progression, an increased probability of recurrence and metastasis, and the deterioration of the prognosis 13,14. In breast cancers, IGFBP-3 has the capability to promote tumour growth through either IGF-1-dependent or IGF-1-independent pathways 12,15. IGFBP3 not only contributes to the sphingosine-1-phosphate (S1P)-dependent transactivation of epidermal growth factor receptor (EGFR) by stimulating sphingosine kinase (SPHK) but also provokes autophagy-related survival through the interaction between IGFBP3 and GRP78 15,16. More importantly, increased expression of the IGFBP3 mRNA and protein in ccRCC has been described in some clinical investigations, while kidney specimens without cancer show little or no expression of this molecule 11,17. According to the results of *in vitro* experiments, IGF-1 treatment of Caki-2 cells (human ccRCC cell line) upregulates IGFBP3, and cell proliferation driven by IGF-1 is obviously increased by exogenous IGFBP3 18,19. Meanwhile, severe combined immunodeficient (SCID) mice that were continuously infused with IGF-1 early after Caki tumour cell inoculation exhibited higher intratumour IGFBP3 expression, a larger tumour size and a higher microvascular density, providing additional evidence that the upregulation of IGFBP3 may be involved in tumour viability and growth 20. Taken together, strategies targeting IGFBP3 and its related signalling pathways may represent a ground-breaking approach to treat ccRCC.

**Figure 1:**
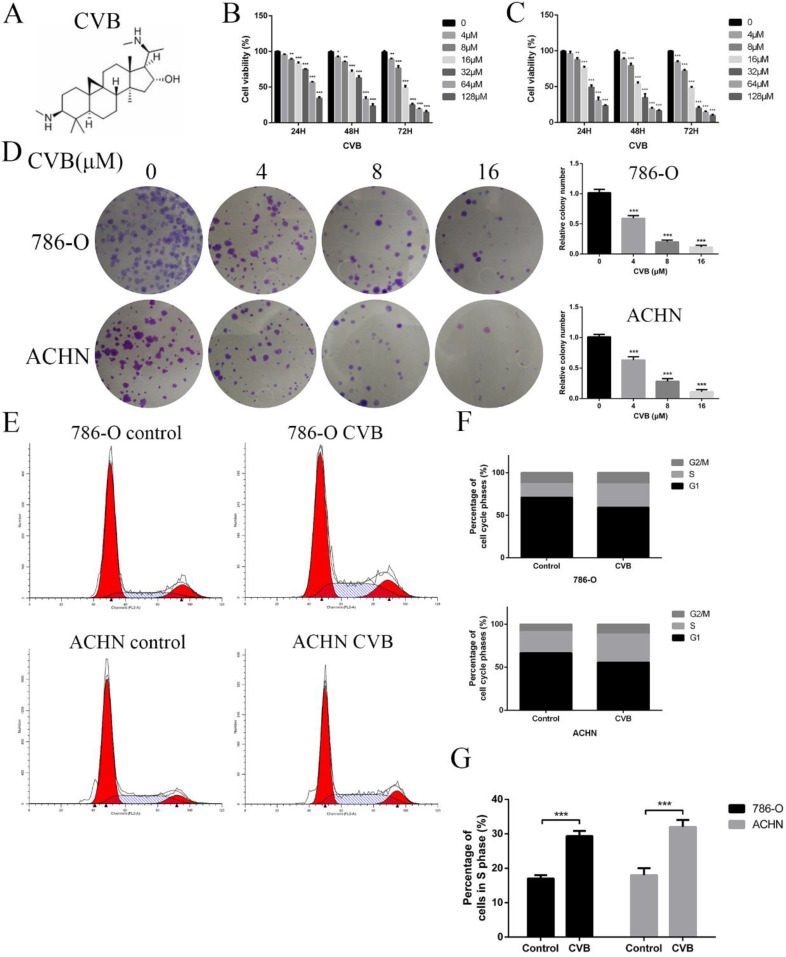
**CVB inhibits the proliferation of ccRCC cells and induces S phase cell cycle arrest. (A)** The chemical structure of CVB. **(B, C)** 786-O and ACHN cells were treated with different concentrations of CVB for 24, 48, and 72 h, and the cell viability was determined by an MTT assay. ^*^P < 0.05, ^**^P < 0.01, ^***^P < 0.01 vs. control group. **(D)** 786-O and ACHN cells were treated with CVB (0, 4, 8, and 16 μM) for 12 h, and the anti-proliferation effect of CVB was detected by a colony formation assay. **(E-G)** The cell cycle distribution was evaluated by flow cytometry. ^***^P < 0.01 vs. control group.

In the past few years, numerous studies have been conducted to develop natural herbal products or phytochemicals that possess antitumour activity and low toxicity. Encouragingly, some plant alkaloids inhibit tumour proliferation, invasion, and metastasis 21-23. Cyclovirobuxine (CVB; Fig. 1A), a steroidal alkaloid component extracted from the roots of the traditional Chinese medicinal herb *Buxus microphylla*, has been used extensively for the prevention and treatment of coronary heart disease, angina pectoris, arrhythmias, heart failure, cerebrovascular diseases and other cardiovascular disorders and to ameliorate insomnia symptoms 24,25. Moreover, Jie Wu et al. reported that CVB inhibits cell proliferation and induces mitochondria-mediated apoptosis in gastric cancer cells 26. Another *in vitro* study also illustrated that CVB promotes autophagy-associated cell death via the AKT/mTOR signalling pathway in human breast cancer cells 27. However, no study has identified the therapeutic efficacy and the underlying molecular mechanism of CVB in RCC. In the present study, we aimed to evaluate the potential anti-RCC effects of CVB *in vivo* and *in vitro*. CVB had a great capacity to inhibit growth, proliferation, angiogenesis, invasion and migration and promote apoptosis in two RCC cell lines. Furthermore, CVB inhibited the invasion and migration of renal carcinoma cells by interfering with the EMT through the suppression of the IGFBP3-AKT/MAPK/STAT3-Snail signalling pathway.

## Materials and methods

### Cells and reagents

The human ccRCC cell lines (786-O and ACHN) were donated by professor Zhang cheng from the first affiliated hospital of Harbin medical university. Human umbilical vein endothelial cell line (HUVECs) and human normal hepatic cell line (LO2) were donated by the Central Laboratory of the First Affiliated Hospital of Harbin Medical University. Human renal tubular epithelial cell line (HK-2) was purchased from the Shanghai Saibaikang Biological Technology Co, Ltd (Shanghai, China). CVB (purity ≥ 99%, cat. no. N117989, Aladdin Industrial Corporation, Shanghai, China) was dissolved to a final concentration of 300 mmol/L in methanol and stored at 4 °C as the stock solution. IGFBP3 was purchased from Sigma-Aldrich (St. Louis, MO, USA). MK2206 and SC-79 were obtained from Topscience biochemical technology. Primary antibodies used to detect Slug, Twist, ZEB1, phospho-ERK, phospho-JNK and phospho-P38 were supplied by Santa Cruz Biotechnology (Santa Cruz, CA, USA). Primary antibodies for detecting phospho-STAT3 (Tyr705), phospho-Akt (Ser473), STAT3, AKT and Vimentin were acquired from Cell Signaling Technology (Beverly, MA, USA). Anti-E-cadherin, anti-IGFBP3, anti-Bcl-2, anti-Bax, and anti-N-cadherin antibodies were obtained from Proteintech Group (Wuhan, China). Anti-Ki67, anti-ERK, anti-JNK, anti-P38 and anti-snail antibodies were supplied by Wanlei Biological Technology Co., Ltd. (Shenyang, China). The antibody against β-actin was procured from ZhongShan Golden Bridge Bio Co., Ltd. (Beijing, China). The horseradish peroxidase conjugated goat anti-mouse and goat anti-rabbit secondary antibodies were obtained from ZhongShan Golden Bridge Bio Co., Ltd. (Beijing, China).

### Cell viability assays

The cytotoxic activity of CVB was detected by using the MTT assay. After 24 h of incubation, cells were treated with various doses of CVB (0-128 μM) for 24, 48 and 72 h. MTT solution (5 mg/ml) was then added into each well for 4 h at 37 °C. Subsequently, the culture medium containing the MTT reagent was removed, and DMSO was added to the cells to dissolve the formazan crystals. The absorbance at 490 nm was then determined with a microplate reader (ELx808, BioTek Instruments, Winooski, VT, USA).

### Colony formation assay

786-O and ACHN (1000 cells/well) cells were seeded into 6-well plates. After an incubation of 24 hours, cells were treated with CVB (0-16 μM) for 12 h, and then the supernatant was changed to complete culture medium. After culturing for 14 days, the cells were first fixed with methanol and then stained with 0.5% crystal violet for 10 min at room temperature. The numbers of cell colonies were counted using a light microscope.

### Cell cycle analysis

ccRCC cells were treated with CVB (40 μM) in a 6-well plate for 48 h. Cells were then collected, washed twice in cold PBS and fixed in 70% ethanol at 4 °C overnight. The next day, the fixed cells were washed with PBS again and then stained with propidium iodide (PI) (40 µg/ml) and RNase A (2.5 mg/ml, 4A Biotech, Beijing, China) at 37 °C in the darkroom for 30 min. A flow cytometer (BD Biosciences, Franklin Lakes, NJ, USA) was used to detect the cell cycle distribution. The assays were repeated three times.

### Cell apoptosis assay

Briefly, after incubation with CVB (0-60 μM) for 48 h, the adherent and floating ccRCC cells were harvested with trypsin and washed with PBS. These washed cells were then collected by centrifugation and resuspended in staining buffer containing 5 µl of annexin V and 5 µl PI. Following incubation of the cells at room temperature for 5 min in the dark, the proportions of cells at different apoptotic phases were identified by flow cytometry.

### Wound healing assays

To conduct the wound healing assays, cells were plated on 6-well plates and grown to 90% confluence. With the help of a 10-μl pipette tip, the confluent monolayer ccRCC cells were scraped linearly to create an artificial scratch. Subsequently, PBS was added to the wells to wash and remove floating cells and cell debris. The cells were then exposed to medium supplemented with CVB (0-16 μM) for 24 h. Thereafter, cell migration was observed by capturing the images at 0 h and 24 h after wounding. ImageJ software (National Institutes of Health, Bethesda, MD, USA) was utilized to measure and analyse the migratory distance.

### Transwell invasion assay

Transwell chambers (8-μm pore size, Corning, Tewksbury, USA) were used to evaluate the migration and invasion abilities of ccRCC cells. First, ccRCC cells stimulated with CVB (0-16 μM) for 24 h were prepared into a cell suspension using serum-free medium. Afterwards, the upper chamber was planted with pre-treated ccRCC cells (3×10^4^ cells/well), while the corresponding lower chamber was filled with 10% FBS medium. For the invasion assay, the upper transwell chambers were precoated with 50 μl of Matrigel (Becton Dickinson, Franklin Lakes, NJ) for 4 h. After 48 h of incubation, the cells on the upper side of the filter were removed by using a cotton swab, and the cells attached to the lower surface of the filter were fixed with methanol for 20 min at room temperature and then stained with 0.5% crystal violet for 20 min at room temperature. Finally, light microscopy (IX51, Olympus, Tokyo, Japan) was used to randomly select 5 visual fields from each sample for photos and the number of cells was calculated.

### Protein extraction and Western blotting analysis

ccRCC cells treated with different concentrations of CVB (0-60 μM) for 48 h were harvested and then lysed using ice-cold RIPA buffer (Beyotime, Shanghai, China). The protein concentration in lysis buffer was determined using the BCA Protein Assay Kit (Beyotime, Shanghai, China). Equal amounts of protein (40 μg) were separated by 10% SDS-PAGE (Beyotime, Shanghai, China) and then electroblotted onto PVDF membranes (Millipore, Bedford, MA). Then, the membranes were blocked with 5% skim milk for 2 h at room temperature and were incubated with specific primary antibodies overnight at 4 °C. Subsequently, the membranes were washed with TBST three times and incubated with the corresponding HRP-conjugated secondary antibody for 2 h at room temperature. The protein signals were visualized with the enhanced chemiluminescence reagent (Wanlei, Shenyang, China). β-actin was used as an internal control.

### Immunofluorescence

Following the abovementioned treatment, the cells were fixed with 4% paraformaldehyde for 20 min at room temperature and permeabilized with 0.5% Triton X-100 for 20 min at room temperature. Subsequently, the coverslips were incubated with 1% bovine serum albumin (BSA; Beyotime, Shanghai, China) for 1 h at room temperature and further incubated with appropriate primary antibodies targeting E-cadherin, Vimentin or Snail overnight at 4 °C. After that, cells were incubated with a fluorescein isothiocyanate-conjugated secondary antibody (ZhongShan Golden Bridge Bio Co., Ltd., Beijing, China) for 1 h in the darkroom and washed with PBS three times. The cells were then stained with DAPI (Beyotime, Shanghai, China) for 5 min to visualize the nucleus and were observed under a fluorescence microscope (BX53, Olympus, Tokyo, Japan) to acquire images. The assays were repeated three times.

### Bioinformatics analysis

To determine the subcellular localization of IGFBP3, the Human Protein Atlas (http://www.proteinatlas.org) was searched. Moreover, the GEPIA (http://gepia.cancer-pku.cn/index.html) database was used to compare the expression level of IGFBP3 in normal tissues and in ccRCC tissues, to understand the changes of IGFBP3 in various kinds of tumors and to perform patient survival analysis.

### Molecular docking

AutoDock software was used to analyze the molecular docking between small molecule CVB and large molecule IGFBP3, and then docking molecule was visualized by pymol software. The intermolecular docking ability is mainly determined by the absolute value of binding energy, in which less than 4 means that the binding ability is very low, between 4 and 5 means that the binding ability is low, between 5 and 7 means that the binding ability is medium, and greater than 7 means that the binding ability is strong.

### IGFBP3 siRNA transfection

IGFBP3 siRNA (50 nM, GenePharma, Shanghai, China) or control siRNA (50 nM, GenePharma, Shanghai, China) were used to transfect cells with the aid of Lipofectamine 2000 (Invitrogen, Carlsbad, California, USA). After transfection for 4-6 h, the medium containing siRNA was changed to fresh complete medium with or without CVB. Forty-eight hours later, cells were collected for Western blotting and MTT assays. The sequences for IGFBP3 and control siRNA are shown in Additional file 1: Table S1.

### Mouse xenograft model and treatments

All animal procedures were performed with the review and approval of the Animal Care and Use Committee. 786-O ccRCC cells at a density of 5×10^6^ cells/200 μl PBS were subcutaneously injected into the flanks of mice. One week after injection, 786-O tumor-bearing mice were randomly divided into the control or CVB treatment group (n = 6, each group). The mice in the CVB treatment group were intraperitoneally injected with CVB at a dose of 0.5 mg/kg daily for 3 consecutive weeks, while the mice in the control group were intraperitoneally injected with sterile saline daily for the same time. The body weights of the mice were measured every 7 days during the treatment. Tumor dimensions were measured using a digital calliper, and the tumor volume was calculated using the following formula: V = length × width^2^/2. The tumor changes were observed by ultrasonic imaging. Before sacrificing, all animals were intraperitoneally anesthetized with sodium pentobarbital (1%, 5 mL/kg). And the final tumor specimens were resected and weighed. The tumor tissues were divided into two parts, one for immunohistochemistry and the other for Western blot analysis.

### Immunohistochemistry (IHC)

Briefly, tumor tissues were fixed with 4% paraformaldehyde, embedded in paraffin and then sliced into 5-μm paraffin sections. After blocking endogenous peroxidase activity with 3% hydrogen peroxide, the sections were treated with 10% goat serum (Solarbio, Beijing, China) for 15 min at room temperature to block nonspecific antigens. Subsequently, the sections were incubated with an anti-IGFBP3, anti-Ki-67, anti-Snail or anti-Vimentin antibody overnight at 4 °C. After treating with diaminobenzidine substrate solution (Solarbio, Beijing, China) and counterstaining with haematoxylin, the slides were dehydrated in the graded ethanol solution and sealed with neutral balsam. The expression of these target proteins was observed and photographed by an independent pathologist using a light microscope at 200× magnification.

### Statistical analysis

Statistical analyses were performed utilizing IBM SPSS 22.0 software (Armonk, NY, USA). Data were expressed as the mean ± standard deviation (SD). Differences between two groups were analysed by Student's t-test, while comparisons among multiple groups were conducted by ANOVA. *P* < 0.05 was considered to indicate statistical significance.

## Results

### CVB reduces the viability and proliferation of human ccRCC cell lines

786-O and ACHN cells were exposed to a wide range of CVB concentrations (0-128 μM) for 24, 48, and 72 h to investigate the effect of CVB on the viability of human ccRCC cells. The results of the MTT assays indicated that CVB noticeably reduced the viability of two ccRCC cell lines in a dose- and time-dependent manner, suggesting that CVB has the ability to suppress the proliferation of ccRCC cells (Fig. 1B and C). According to the probit regression analysis, the 50% inhibitory concentration values (IC50) of CVB in 786-O and ACHN cells were 25.8 μM and 40.5 μM at 48 h, respectively. Additionally, CVB exerted a negligible effect on human renal tubular epithelial cells (HK-2), HUVECs and human normal hepatic cells (LO2) (Fig. S1A). A colony formation assay was also performed to evaluate the anticancer effect of CVB, and the number of clonogenic CVB-treated cells was significantly decreased compared with the number of control cells (Fig. 1D). Deregulation of the cell cycle plays an important role in the excessive and rapid proliferation of tumour cells. As shown in Fig. 1E-G, the stimulation of cells with CVB for 48 h led to an increased accumulation of ccRCC cells in S phase and a corresponding decrease in the number of cells in G1 phase. Taken together, these findings provide sufficient evidence that CVB suppresses the proliferation of 786-O and ACHN cells.

### CVB induces apoptosis in ccRCC cells

**Figure 2:**
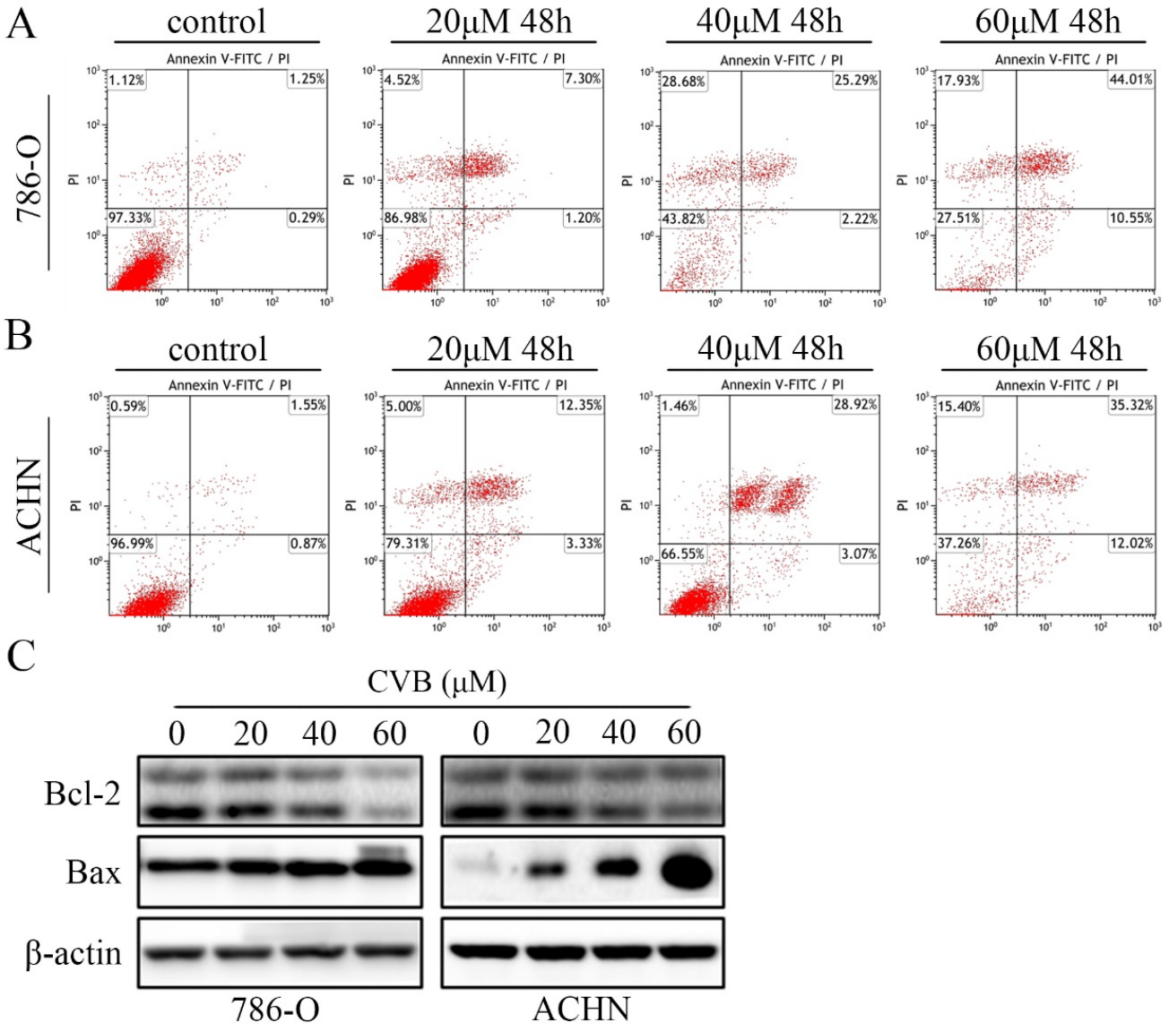
**CVB induces apoptosis in ccRCC cells. (A, B)** Following treatment with CVB (0, 20, 40, and 60 μM) for 48 h, 786-O and ACHN cells were stained with annexin V-FITC/PI to detect apoptotic cells by flow cytometry. **(C)** The expression of Bcl-2 and Bax was analysed by Western blotting. β-actin was used as a loading control.

Because the CVB-treated cells exhibited distinct morphological changes and the induction of apoptosis was one of the critical factors contributing to the inhibition of cell proliferation, ccRCC cells stained with annexin V and PI were subjected to flow cytometry. As indicated in Fig. 2A and B, treatment with a series of different concentrations of CVB resulted in apparent dose-dependent apoptosis in 786-O and ACHN cells. The apoptotic indices were 2.51, 9.00, 29.57 and 54.23 in 786-O cells and 3.13, 12.03, 31.33 and 50.90 in ACHN cells treated with 0, 20, 40 and 60 μM CVB, respectively (Fig. S1B). Furthermore, the proverbial apoptosis regulatory proteins were examined using Western blot analysis to understand the mechanism by which CVB induces cell apoptosis. As expected, CVB dose-dependently decreased the expression of Bcl-2 (an antiapoptotic protein) and increased the expression of Bax (a proapoptotic protein) (Fig. 2C). Based on these results, the antitumour effects of CVB are mediated by promoting cell apoptosis to some extent.

### CVB inhibits the angiogenesis, migration and invasion of ccRCC cells

**Figure 3:**
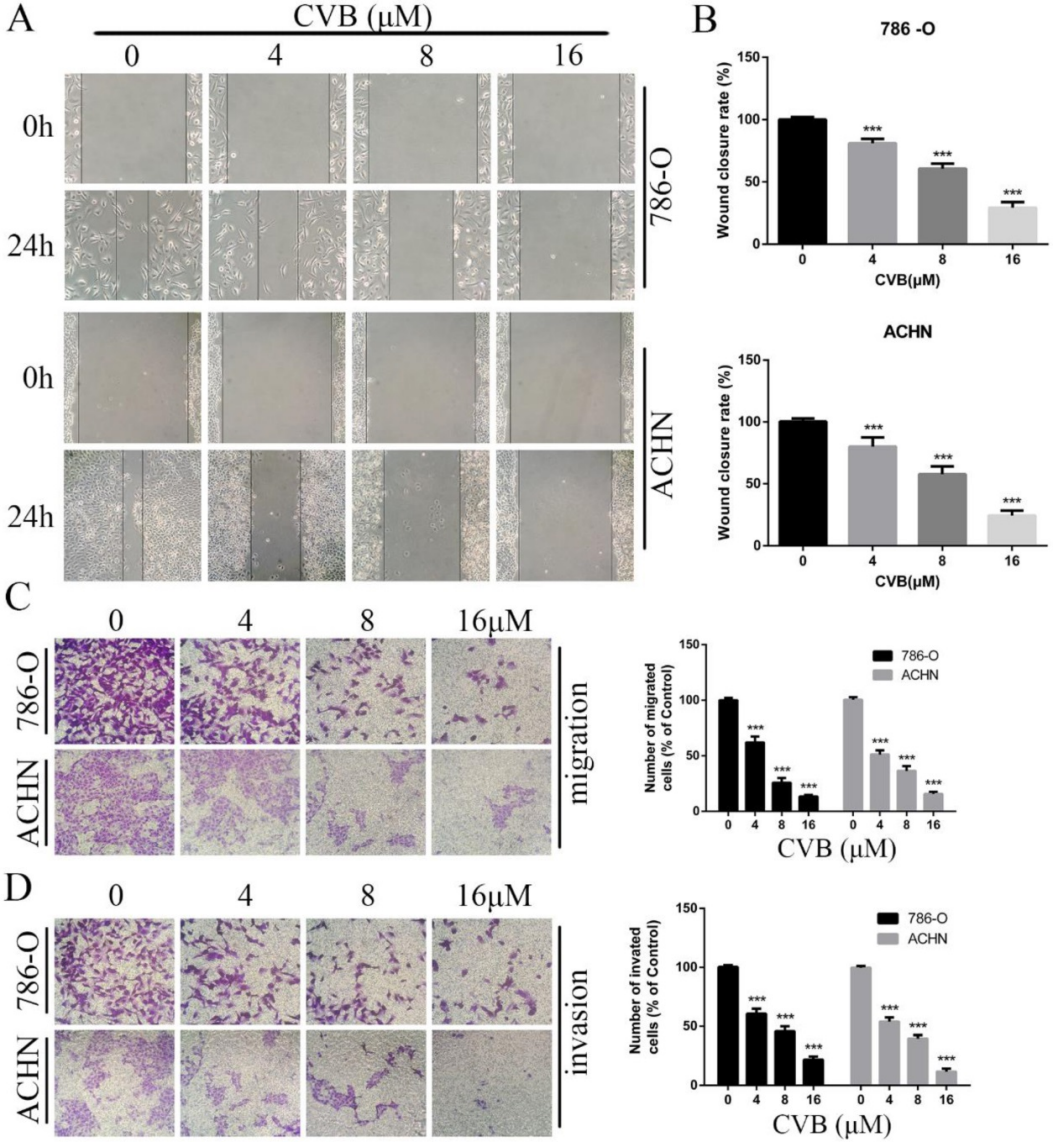
**CVB inhibits the migration and invasion of ccRCC cells. (A, B)** 786-O and ACHN cells were treated with CVB (0, 4, 8, and 16 μM) for 24 h and then subjected to wound healing assays. ^***^P < 0.01 vs. control group. **(C, D)** 786-O and ACHN cells were treated with CVB (0, 4, 8, and 16 μM) for 24 h, and the migratory and invasive abilities were assessed. ^***^P < 0.01 vs. control group.

Since migratory and invasive features are hallmarks of renal cancer, the pharmacological effect of CVB on ccRCC cells was determined using wound healing and Transwell assays. As indicated in Fig. 3A, B and C, the number of migrated cells was obviously reduced after treatment with CVB in a dose-dependent manner, with the strongest inhibition observed for 16 μM CVB. Additionally, compared with control cells, CVB-treated cells displayed damaging invasive characteristics, and the extent of invasion was positively correlated with the CVB concentration (Fig. 3D). In conclusion, the inhibitory effect of CVB on RCC is partially attributable to impeding the migration and invasion of ccRCC cells.

### CVB suppresses the EMT in human ccRCC cells

**Figure 4:**
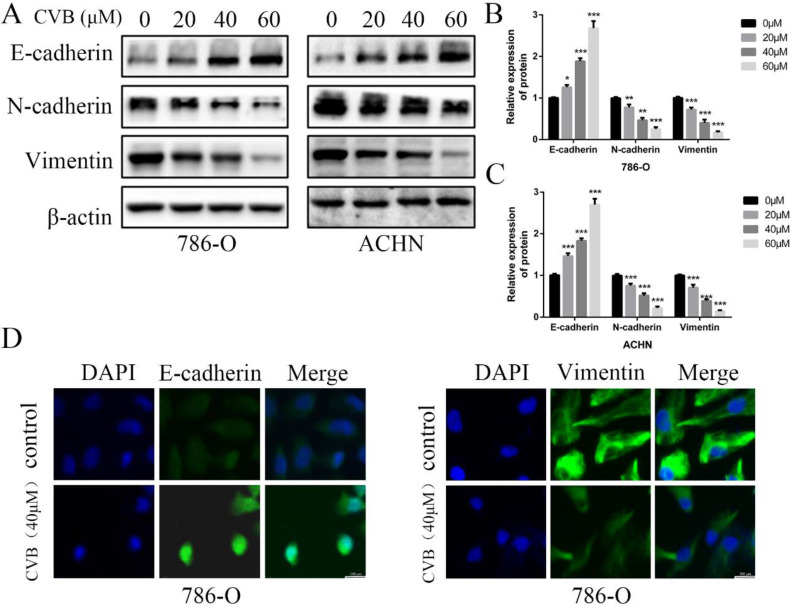
**CVB suppressed the EMT process in human ccRCC cells. (A-C)** 786-O and ACHN cells were treated with CVB (0, 20, 40, and 60 μM) for 48 h. Western blotting was conducted to detect the protein levels of the EMT-related markers E-cadherin, N-cadherin and vimentin. β-actin was used as a loading control. ^*^P < 0.05, ^**^P < 0.01, ^***^P < 0.01 vs. control group. **(D)** After treatment with 20 μM CVB for 48 h, the expression and distribution of E-cadherin and vimentin were examined by immunofluorescence. Original magnification: 200×.

The EMT has been considered an indispensable biological step for endowing cancer cells with stronger migratory and invasive capacities. Therefore, the expression of well-known EMT-related biomarkers was examined using Western blot analysis to ascertain whether CVB modulated cell migration and invasion by affecting the EMT in ccRCC cells. As presented in Fig. 4A-C, the mesenchymal markers N-cadherin and Vimentin were markedly downregulated by increasing concentrations of CVB. However, the epithelial marker E-cadherin was upregulated after treatment with CVB. In addition, immunofluorescence staining for E-cadherin and Vimentin produced similar results to the protein levels (Fig. 4D). Collectively, these data clearly confirm that CVB plays a role in suppressing renal tumours by preventing the EMT.

### Snail expression is inhibited by CVB in ccRCC cells

**Figure 5:**
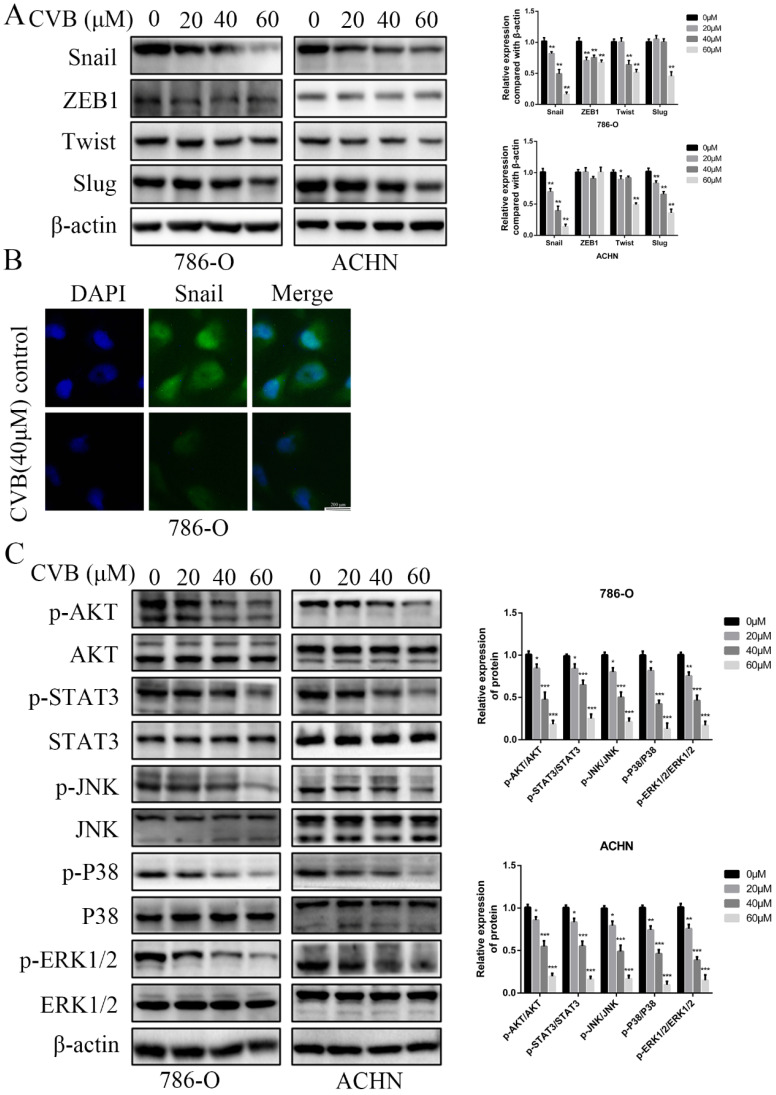
**CVB inhibits the expression of Snail and the activation of the AKT/STAT3/MAPK signalling pathway. (A)** CVB treatment (0-60 μM, 48 h) resulted in a dose-dependent reduction in the EMT regulatory factor Snail. ^*^P < 0.05, ^**^P < 0.01. **(B)** Immunofluorescence results of Snail after CVB treatment (20 μM, 48 h) in 786-O cells. Scale bar: 200 μm. **(C)** After CVB treatment (0-60 μM, 48 h), the expression levels of signalling pathway proteins (the phosphorylated and total proteins of AKT, STAT3, JNK, P38, and ERK) were analysed by Western blotting. β-actin was used as a loading control. ^*^P < 0.05, ^**^P < 0.01, ^***^P < 0.01 vs. control group.

Several crucial EMT-related transcription factors were assessed at the same time to obtain a better understanding of the molecular mechanisms by which CVB regulates the EMT. As depicted in Fig. 5A, CVB treatment resulted in a dose-dependent reduction in the expression of Snail in both 786-O and ACHN cells, concomitant with comparatively irregular changes in the expression of ZEB1, Twist and Slug. Immunofluorescence staining further confirmed that Snail expression was diminished in CVB-treated cells (Fig. 5B). Moreover, emerging studies have reported that Snail is upregulated in some cancers, including ccRCC, and manipulates cell survival, proliferation, apoptosis, angiogenesis and the EMT. Based on these findings, we concluded that the reduction in Snail expression may be an important component of the mechanism by which CVB blocks tumour growth, migration and invasion.

### CVB represses the activities of ccRCC cells by inhibiting the AKT/STAT3/MAPK signalling pathway

The PI3K/AKT, JAK/STAT3 and MAPK signalling pathways are the three common axes regulating many different cellular activities, such as cell survival, proliferation, apoptosis, transformation and death. To elucidate the underlying mechanism of the distinct inhibition of ccRCC cells induced by CVB, The levels of total and phosphorylated AKT, STAT3 and the major members of the MAPK family (JNK, P38 and ERK) were detected using Western blot analysis to elucidate the mechanism underlying the distinct CVB-induced inhibition of ccRCC cell activity. As shown in Fig. 5C, exposure to increasing doses of CVB robustly reduced the p-AKT, p-STAT3, p-JNK, p-P38 and p-ERK levels in renal cancer cells in a dose-dependent manner. However, the expression of AKT, STAT3, JNK, P38 and ERK in cells treated with CVB was almost unchanged compared with that in the control cells. Because previous studies have reported that the AKT pathway is the most critical pathway in renal cell carcinoma, an AKT inhibitor (MK2206) was used to investigate whether CVB exerts antitumour effects mainly through the AKT signalling pathway. As shown in Fig. S2A, both CVB and the AKT inhibitor (MK2206) markedly reduced the viability of ccRCC cells. More importantly, the combination of CVB and MK2206 did not further enhance the inhibitory effect compared to treatment with CVB or MK2206 alone, and the addition of AKT agonist SC-79 antagonized the effects of MK2206 and CVB on cell activity. Based on these results, the AKT-dependent pathway is extremely important for the antitumour effect of CVB. In summary, CVB efficiently represses the activities of ccRCC cells by inhibiting the AKT/STAT3/MAPK signalling pathway.

### IGFBP3 is an indispensable therapeutic target of CVB in ccRCC cells

**Figure 6:**
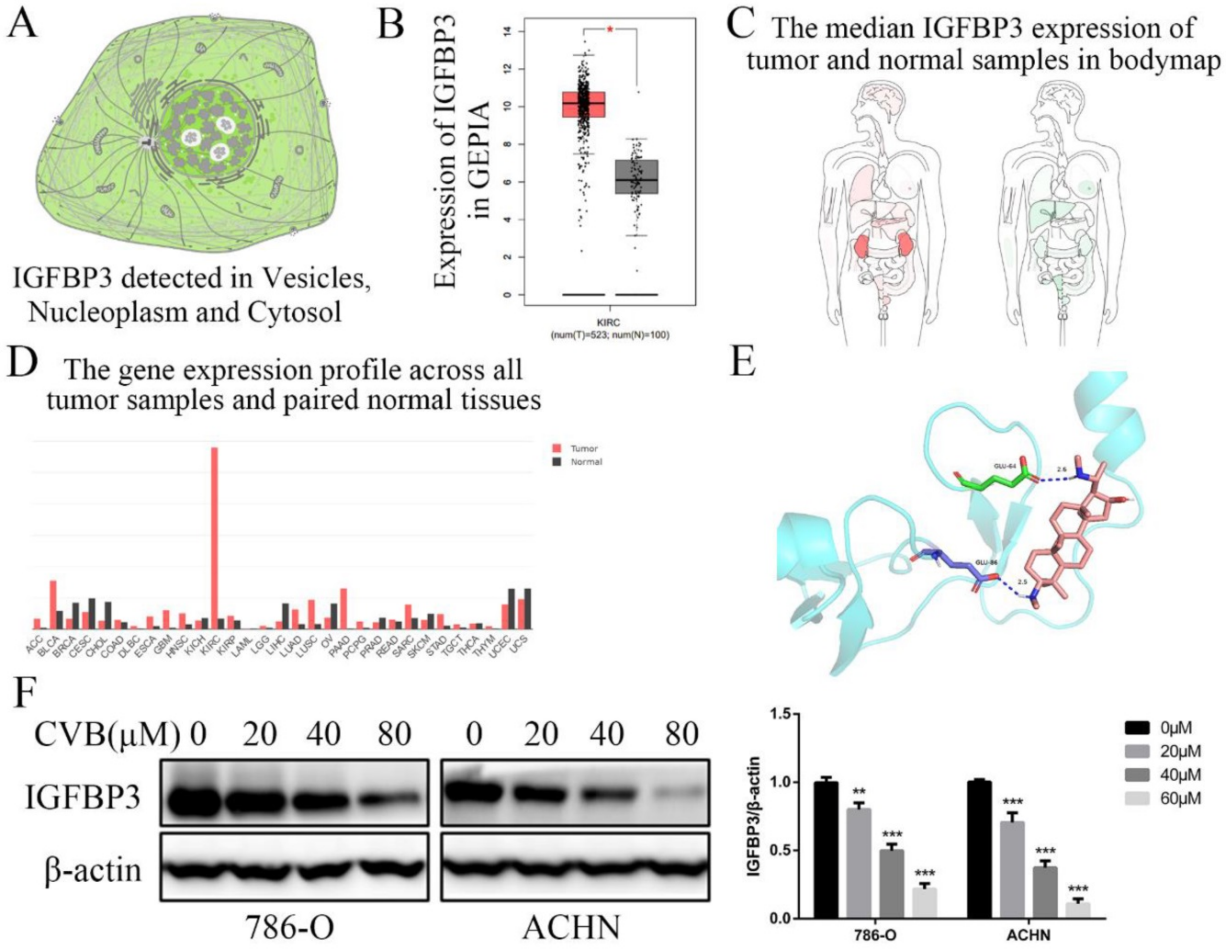
**IGFBP3 is the critical therapeutic target for CVB in ccRCC cells. (A)** Distribution of IGFBP3 in cells (the HPA). **(B)** The mRNA level of IGFBP3 in healthy kidney tissues and RCC (GEPIA). **(C)** The median IGFBP3 expression of tumour (red) and normal (green) samples in Body Map (GEPIA). **(D)** The IGFBP3 mRNA levels among multiple kinds of common tumours (GEPIA). **(E)** Protein structure conformation between CVB and IGFBP3. **(F)** The protein level of IGFBP3 in 786-O and ACHN cells that were treated with 0, 20, 40, and 80 μM CVB for 48 h. β-actin was used as a loading control. ^**^P < 0.01, ^***^P < 0.01 vs. control group. **(G)** Immunofluorescence was performed to detect IGFBP3 in 786-O and ACHN cells treated with CVB (20 μM, 48 h). Original magnification: 200×.

**Figure 7:**
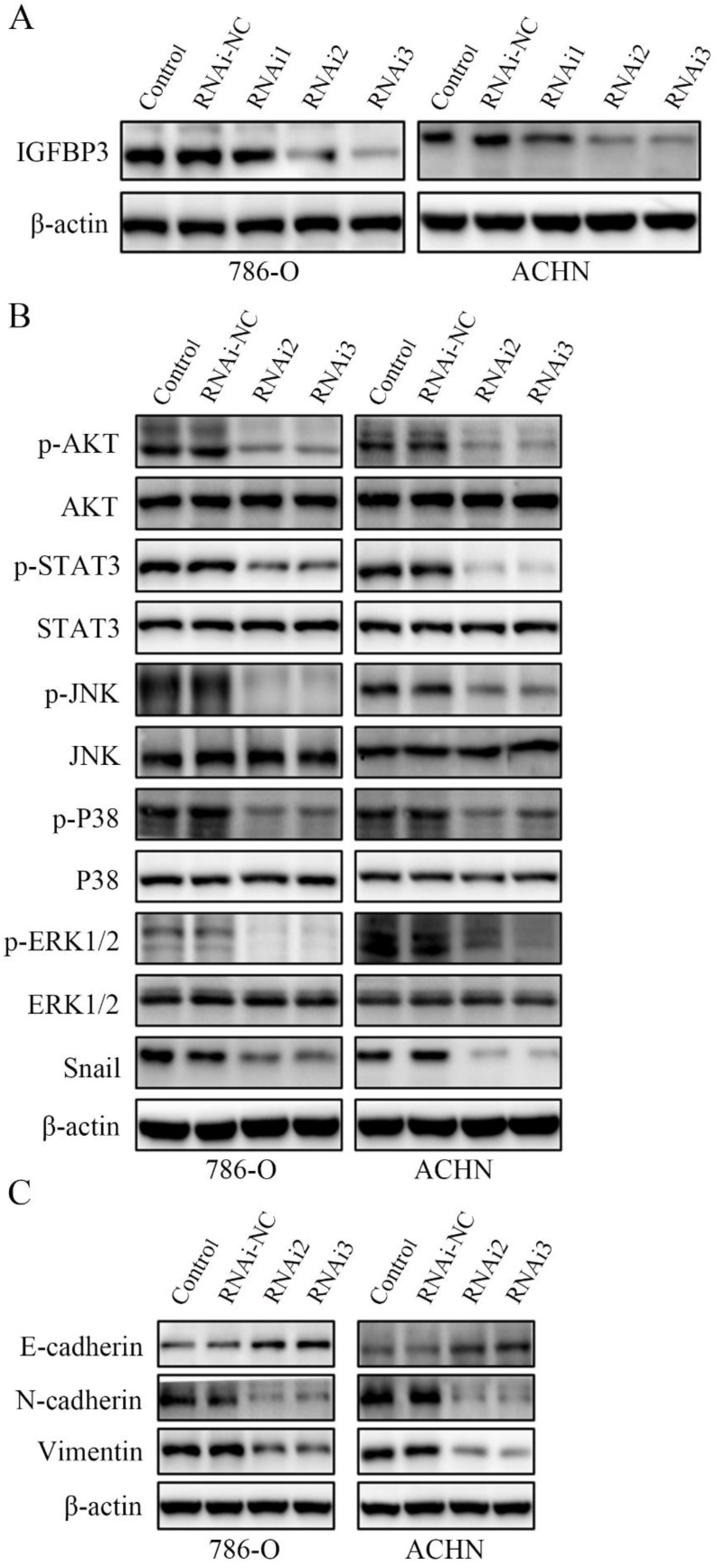
**IGFBP3 is the upstream factor of the AKT/STAT3/MAPK-Snail signalling pathway. (A)** IGFBP3-targeting siRNA RNAi2 and RNAi3 effectively silenced IGFBP3 in 786-O and ACHN cells. **(B)** IGFBP3-targeting siRNA RNAi2 and RNAi3 resulted in an obvious inhibition of the AKT/STAT3/MAPK-Snail signalling pathway. **(C)** IGFBP3 siRNA alleviate EMT in ccRCC cells.

**Figure 8:**
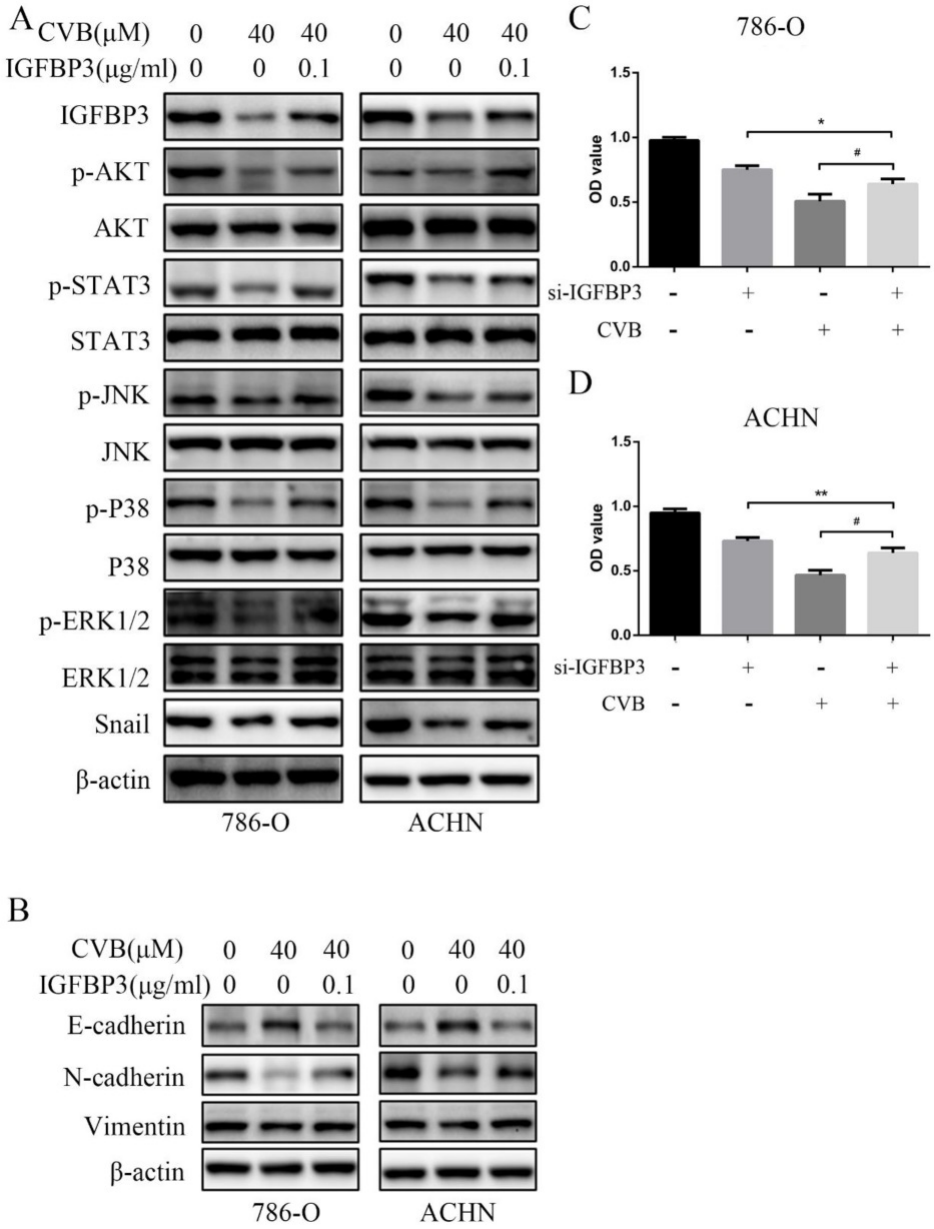
**IGFBP3 is the dominant therapeutic target of CVB. (A)** Exogenous IGFBP3 significantly abolished the inhibitory effect of CVB on the activation of AKT/STAT3/MAPK-Snail signaling pathway. **(B)** Exogenous IGFBP3 significantly abolished the inhibitory effect of CVB on EMT.** (C)** CVB-induced (40 μM, 48 h) growth inhibition of 786-O cells was dependent on the suppression of IGFBP3. ^*^P < 0.05 vs. si-IGFBP3 group.^ #^*P*< 0.05 vs. CVB group. **(D)** CVB-induced (40 μM, 48 h) growth inhibition of ACHN cells was dependent on the suppression of IGFBP3. ^**^P < 0.01 vs. si-IGFBP3 group.^ #^*P*< 0.05 vs. CVB group.

IGFBP3 was observed in vesicles, the neoplasm and cytosol in the Human Protein Atlas (HPA) (Fig. 6A). According to the accumulated GEPIA data, renal tumour tissues expressed higher IGFBP3 levels than normal renal tissues (Fig. 6B and C). In addition, among various common tumours, IGFBP3 mRNA levels were increased most significantly in renal cell carcinoma (Fig. 6D). More importantly, the patients in the low IGFBP3 groups experienced longer disease-free survival (DFS) than those in the high IGFBP3 groups (Fig. S2C). Meanwhile, the results of the analysis of the spatial conformation of IGFBP3 showed that the binding energy of molecular docking between CVB and IGFBP3 is -7.29 kcal/mol, indicating very strong binding. The oxygen atom of CVB interacts with two amino acid residues (GLU-64 and GLU-86) of IGFBP3 to form hydrogen bonds (Fig. 6E). Because IGFBP3 was overexpressed in renal cancers and identified as a driving force for other tumours, the next question was how IGFBP3 functions in ccRCC and whether it is the target of CVB therapy. First, IGFBP3 expression was reduced in a dose-dependent manner following CVB treatment, with the maximal suppression observed in cells treated with 80 μM CVB (Fig. 6F). Then, a specific siRNA was transfected into ccRCC cells to downregulate IGFBP3 and determine its biological function. As indicated in Fig. 7A and B, RNAi2 and RNAi3 not only significantly silenced IGFBP3 but also reduced the levels of p-AKT, p-STAT3, p-JNK, p-P38, p-ERK and Snail. Although no evident changes in the total AKT, STAT3, JNK, P38 and ERK levels were observed, these results still suggest that IGFBP3 may be upstream of the AKT/STAT3/MAPK-Snail signalling pathways. Moreover, IGFBP3 knockdown led to the upregulation of E-cadherin and downregulation of N-cadherin and Vimentin (Fig. 7C), indicating that IGFBP3 silencing alleviated the EMT in ccRCC cells. Subsequently, exogenous IGFBP3 was added to CVB-treated ccRCC cells to ascertain whether IGFBP3 was a therapeutic target of CVB. IGFBP3 supplementation almost completely abolished the inhibitory effect of CVB on the activation of the AKT/STAT3/MAPK-Snail signalling pathway and the occurrence of the EMT (Fig. 8A and B). In addition, both CVB and the IGFBP3 siRNA reduced the proliferation of ccRCC cells. However, the inhibitory effect of CVB was partially reversed by the downregulation of IGFBP3 (Fig. 8C and D). These experiments indicated that IGFBP3 is an indispensable therapeutic target of CVB in ccRCC cells.

### CVB inhibits the growth of 786-O xenograft tumours in a nude mouse model by suppressing the IGFBP3-AKT/STAT3/MAPK-Snail signalling pathway

**Figure 9:**
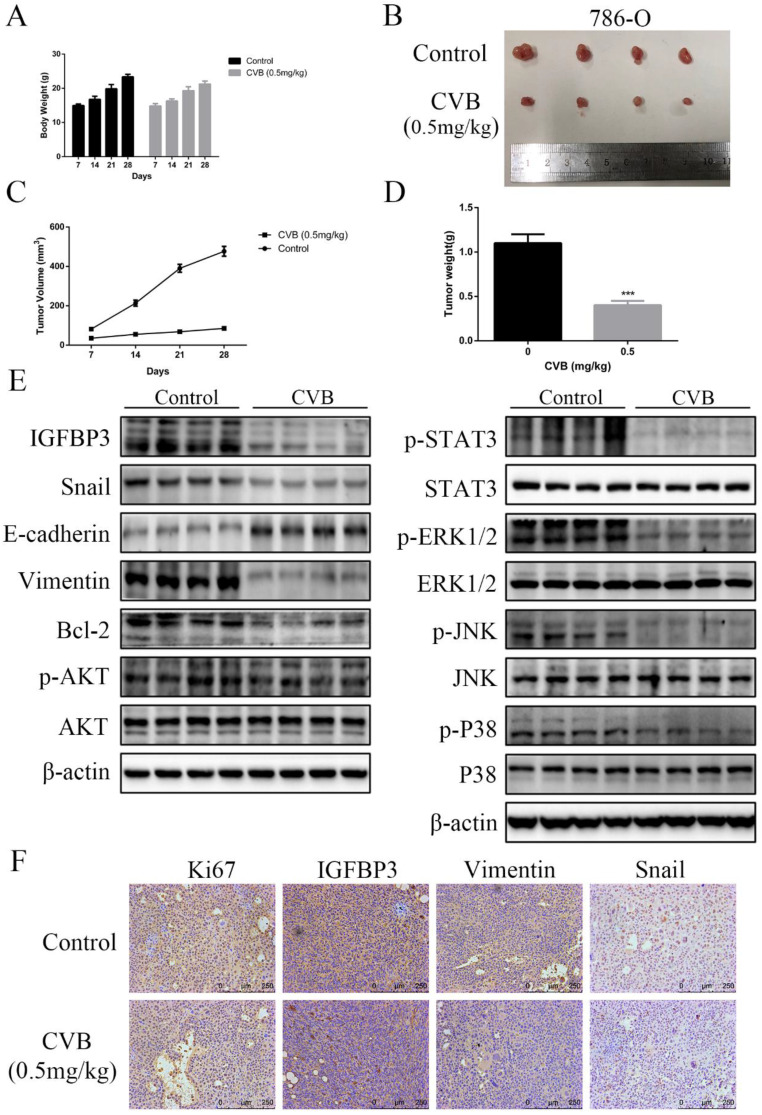
** (A)** The body weights of saline-treated mice and CVB-treated mice were assessed every 7 days. **(B)** Four representative tumors were excised from the control group or the CVB-treated group. **(C)** Tumor volumes were calculated by measuring tumor dimensions with callipers every 7 days. ^*^P < 0.05, ^**^P < 0.01, ^***^P < 0.01 vs. control group. **(D)** The average weight of xenograft tumors. ^**^P < 0.01 vs. control group. **(E)** The protein levels of IGFBP3, the AKT/STAT3/MAPK signaling pathway components, Snail, E-cadherin, Vimentin and Bcl-2 were analysed by Western blot analysis. β-actin was used as a loading control. **(F)** Immunohistochemical analysis was carried out to detect the expression of IGFBP3, Snail, Ki-67, and Vimentin in the control group and the CVB group. Original magnification: 200×.

A mouse xenograft ccRCC model was established to study the antitumour effects of CVB on ccRCC *in vivo*. As shown in Figs. S2B and Fig. 9A-D, a significant difference in body weight was not observed between the two groups, but the administration of CVB effectively reduced the tumour volume and tumour weight. Related signalling pathways and the corresponding cell biological processes involved in the effects of CVB *in vitro* were analysed using Western blotting to further reveal the exact mechanism by which CVB inhibits tumour growth. Compared with the control group, the xenograft tumours from mice treated with CVB exhibited an evident decrease in the levels of IGFBP3, p-AKT, p-STAT3, p-ERK, p-JNK, p-P38, Bcl-2, Snail, and Vimentin (Fig. 9E), while the levels of cleaved Caspase 3 and E-cadherin were increased. In addition, IGFBP3, Snail, Vimentin (EMT-related marker) and Ki-67 (a cell proliferation marker) were analysed using immunohistochemical staining. Similarly, compared with the xenograft tumours from mice injected with the saline solution, the tumour tissues from the CVB-treated mice presented substantially decreased numbers of IGFBP3-, Snail-, Vimentin- and Ki-67-positive cells (Fig. 9F).

## Discussion

RCC has emerged as the genitourinary malignancy with the third highest incidence, and more than 350,000 people worldwide are diagnosed with this disease every year 28. RCC consists of distinct pathological types, of which the major type is ccRCC and the minor type consists of papillary, chromophobe, or oncocytoma 29. Despite remarkable progress in the diagnosis and treatment of RCC over the past few decades, RCC is still one of the top ten tumours, shortening the lifespan of patients due to its general resistance to the existing treatments and poor therapeutic response 30. Therefore, scientists have expressed increasing interest in revealing the aberrant mechanism regulating tumorigenesis and determining efficient therapeutic targets. As the natural active ingredient of *B. microphylla*, CVB exerted noticeable antitumour effects on several types of tumours, such as breast cancer and gastric cancer 26,27. Nevertheless, the exact effect of CVB and its associated mechanisms in RCC remain unclear. Our present study revealed for the first time that CVB inhibits proliferation, the EMT and angiogenesis and promotes apoptosis by modulating the AKT/STAT3/MAPK-Snail signalling pathway through the repression of IGFBP3 in ccRCC cells.

First, normal human cells and renal tumour cells were treated with CVB and subjected to cell viability assays to explore the specific inhibitory effects of CVB on tumour cell growth, and we discovered that CVB only exerted inhibitory effects on renal cancer cells. Meanwhile, the colony formation assay provided additional support that CVB exerted a specific antiproliferative effect on ccRCC cells. Cell cycle distribution assays revealed that CVB caused 786-O and ACHN cells to arrest in S phase. Since the proapoptotic effect is a common universal function of some antitumour agents, we detected changes in cell apoptosis using flow cytometry and found a dose-dependent proapoptotic effect of CVB. In addition, CVB upregulated Bax and downregulated Bcl-2, indicating that CVB-induced growth inhibition is related not only to antiproliferative effects but also to apoptosis induction. Subsequently, we performed wound healing assays and Transwell assays to detect the anti-migration and anti-invasion effects of CVB. CVB successfully restrained the migration and invasion of renal tumour cells. All these results indicate that CVB impairs the growth and spread of ccRCC by regulating various biological processes.

The EMT, the prerequisite biological process for the invasion and migration of tumour cells, has been reported to be associated with stronger proliferative, angiogenetic, and antiapoptotic properties. Importantly, the EMT is closely correlated with the adverse outcomes of patients with tumours 31. Therefore, the effect of CVB on the EMT in ccRCC cells has become the main emphasis of our study. Our results showed significantly decreased expression of N-cadherin and Vimentin, while E-cadherin expression was increased after CVB treatment. Thus, CVB inhibits the migration and invasion of ccRCC cells by repressing the EMT. Since a broad array of studies have documented that the EMT is regulated by several transcription factors, we examined the expression levels of four common regulatory factors, namely, Snail, Slug, Twist and ZEB1. Snail was downregulated in ccRCC cells treated with CVB; however, the changes in the other three transcription factors were very small. Furthermore, Snail enhances the migration and invasion of renal cancer and appears to be a potential prognostic protein marker 32. Based on the facts described above, we can conclude that CVB may exert its anti-ccRCC functions by reducing the expression of Snail. Multiple biological signalling cascades participate in the regulation of Snail, including the PI3K/AKT signalling pathway, JAK/STAT3 signalling pathway and MAPK signalling pathway, and these pathways are expected to be attractive therapeutic targets for ccRCC 33-35. In our study, all of these cascades, without exception, were remarkably inhibited by CVB, indicating that the suppression of AKT/STAT3/MAPK-Snail signalling networks may be the mechanism by which CVB exerts its powerful therapeutic effects on ccRCC. Importantly, the inhibitory effects of CVB and the AKT inhibitor MK2206 alone or in combination on the viability of ccRCC cells were approximately the same, indicating that the AKT-dependent pathway may be the most critical for the antitumour effect of CVB. However, the network relationship between the AKT, STAT3 and MAPK pathways in the presence of CVB requires further study.

IGFBP3, the most extensively researched IGFBP, is involved in oncogenesis through the modulation of multiple complicated pathways. In a study by Scully et al., host IGFBP3 expression accelerated the growth of EO771 murine breast cancer by preventing CD8^+^ T cell accumulation 14. Natsuizaka et al. reported that IGFBP3 facilitates the TGF-β1-mediated EMT and activates transcriptional regulators, such as ZEB1, ZEB2, and Snail, in transformed human oesophageal epithelial cells through an IGF-independent mechanism 36. Another experiment identified a role for IGFBP3 in the growth of oesophageal squamous cell carcinoma cells with high CD44 expression by suppressing ROS production through an IGF-independent mechanism in the hypoxic tumour microenvironment and ultimately exerts tumour-promoting activities 37,38. Moreover, the potentiation of IGF-1-dependent proliferation by IGFBP3 was documented in fibroblasts, as well as some cancer cells 39,40. In 2014, Lin et al. published a profound study delineating that IGFBP3 facilitates cancer cell survival by repairing the DNA double-strand breaks caused by genotoxic stress through an increase the autophosphorylation of DNA protein kinase catalytic subunit (DNA-PKcs) and the formation of nuclear EGFR-DNA-PKcs complexes 41. Moreover, activation of the MAPK/ERK signalling pathway in oral squamous cell carcinoma cells and breast epithelial cells has been linked to the upregulation of IGFBP3 42,43. The PI3K/AKT signalling pathway is directly regulated by endogenous IGFBP3 in MAC-T cells to mediate the function of IGF 44. A previous study in patients with NSCLC found that tumours with high levels of IGFBP-3 displayed more obvious activation of insulin-like growth factor 1 receptor (IGF-1R), indicating that IGFBP3 potentiates the activation of IGF-1R to some extent 45. More importantly, IGF-1R has been reported to mediate the tyrosine phosphorylation of STAT3 *in vitro* and *in vivo* with the assistance of active JAK, reminding us that some relationship may exist between IGFBP3 and the JAK/STAT3 signalling pathway 46. In addition, the GEPIA database showed that IGFBP3 was overexpressed in several malignant tumours, and the highest level was observed in ccRCC. Based on these facts and our findings, we speculate that IGFBP3 may exhibit growth-stimulatory functions in ccRCC and that the inhibitory effect of CVB on ccRCC cells may rely on inhibiting IGFBP3. As expected, the expression of IGFBP3 in ccRCC cells was decreased after CVB treatment, consistent with the changes in p-AKT, p-STAT3, p-JNK, p-P38, p-ERK and Snail levels. Then, we conducted a gene knockdown experiment to eliminate the expression of IGFBP3 using an IGFBP3 siRNA. IGFBP3 knockdown noticeably reduced the levels of p-AKT, p-STAT3, p-JNK, p-P38, p-ERK and Snail, suggesting that IGFBP3 was indeed the pivotal upstream factor of the AKT/STAT3/MAPK-Snail signalling pathways in ccRCC cells. Meanwhile, IGFBP3 downregulation also led to the suppression of the EMT, indicating its tumour-promoting effect. We added IGFBP3 to CVB-treated ccRCC cells to determine whether IGFBP3 is a critical target of CVB. Exogenous IGFBP3 significantly reversed the inhibitory effect of CVB on the activation of the AKT/STAT3/MAPK-Snail signalling pathway and the occurrence of the EMT. In addition, although the IGFBP3 siRNA alone obviously restrained cell proliferation in a manner similar to the effect of CVB, the addition of CVB to ccRCC cells transfected with the IGFBP3 siRNA significantly weakened the inhibitory effect of CVB. Thus, IGFBP3 was the dominant therapeutic target of CVB and the suppression of the IGFBP3-AKT/STAT3/MAPK-Snail signalling pathway may be the underlying mechanism by which CVB exerts its antitumour effects on ccRCC.

Mice bearing human ccRCC xenografts were administered CVB or saline for 3 weeks to provide additional insights into the inhibitory effect of CVB on ccRCC *in vivo* and the relevant molecular mechanism. Our data showed that CVB effectively suppressed xenograft growth, as the tumour volumes and tumour weights in the CVB group were smaller than those in the control group. CVB also decreased the expression of multiple components of the IGFBP3-AKT/STAT3/MAPK-Snail signalling pathway. Additionally, the pattern of changes in the levels of representative apoptotic regulatory proteins (Bcl-2) was consistent with the results of the *in vitro* experiments, indicating that CVB induced apoptosis and suppressed angiogenesis. Meanwhile, we detected the expression levels of EMT-related proteins (Snail, E-cadherin, and Vimentin) using Western blotting and immunohistochemistry and found that CVB inhibited the occurrence and progression of the EMT *in vivo*. Furthermore, compared with the saline-treated mice, the CVB-treated ccRCC tissues contained fewer Ki-67-positive cells, indicating that CVB suppressed cell proliferation. These findings further confirm that CVB blocks the progression of ccRCC by modulating proliferation, EMT, angiogenesis and apoptosis through the inhibition of the IGFBP3-AKT/STAT3/MAPK-Snail signalling pathway.

In summary, to the best of our knowledge, our study is the first to show that IGFBP3 is an oncogenic protein that functions as the common upstream regulator of the AKT, STAT3 and MAPK signalling pathways and the EMT transcription factor Snail in ccRCC. We also present a comprehensive set of evidence from cell-based and animal experiments establishing CVB as an anti-ccRCC drug that impedes proliferation, the EMT, angiogenesis, migration and invasion and accelerates apoptosis through the targeted inactivation of the IGFBP3-AKT/STAT3/MAPK/Snail signalling pathways (Fig. S3). In the future, in-depth investigations and clinical trials are warranted to evaluate the optimal therapeutic dose and efficacy of CVB in patients with ccRCC.

## Supplementary Material

Supplementary figures and tables.

## Abbreviations

RCC: Renal cell cancer
ccRCC: clear cell renal cell carcinoma
CVB: Cyclovirobuxine
EMT: epithelial-mesenchymal transition
IGFBP-3: Insulin-like growth factor binding protein-3
HK-2: human renal tubular epithelial cell
HUVECs: human umbilical vein endothelial cell
LO2: human normal hepatic cell
DFS: disease-free survival
