# Cyclovirobuxine inhibits the progression of clear cell renal cell carcinoma by suppressing the IGFBP3-AKT/STAT3/MAPK-Snail signalling pathway: Erratum

**DOI:** 10.7150/ijbs.87837

**Published:** 2024-12-01

**Authors:** Yadong Liu, Huiyan Lv, Xingyi Li, Jiannan Liu, Song Chen, Yaodong Chen, Yinshan Jin, Ruihua An, Shiliang Yu, Zhigang Wang

**Affiliations:** 1 Institute of Ultrasound Imaging, The Second Affiliated Hospital of Chongqing Medical University, Chongqing 400010, China.; 2 State Key Laboratory of Ultrasound in Medicine and Engineering, Chongqing Medical University, Chongqing 400016, China.; 3 Department of Nephrology, The First Affiliated Hospital of Harbin Medical University, No.23 You Zheng Street, Harbin 150001, Heilongjiang, China.; 4 Department of Ultrasonic Imaging, Ningbo First Hospital, The Affiliated Hospital of Ningbo University, Ningbo, China.; 5 Department of Urology, The First Affiliated Hospital of Harbin Medical University, No.23 You Zheng Street, Harbin 150001, Heilongjiang, China.; 6 Department of Ultrasonic Imaging, First Clinical Medical College, Shanxi Medical University, Taiyuan, 030001, Shanxi Province, China.

In the original version of our article, there was an error in Figure 2A and 2B. Specifically, the main reasons were improper gating during flow data analysis and too few cells collected in the last two groups. Figure S1 is a statistical diagram of apoptosis results. We checked the original data again and made sure that the conclusion of the article was not affected by the error. In this regard, all authors have agreed to the erratum, and we apologize for any inconvenience caused by the negligence in our work.

The correct figures are provided in the following Figure A and Figure B.

## Figures and Tables

**Figure A:**
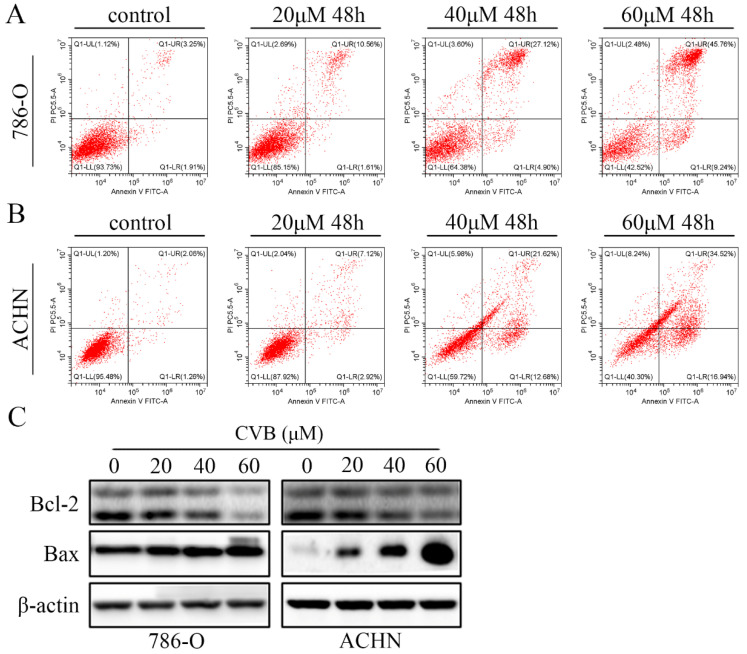
Correct image of Figure 2.

**Figure B:**
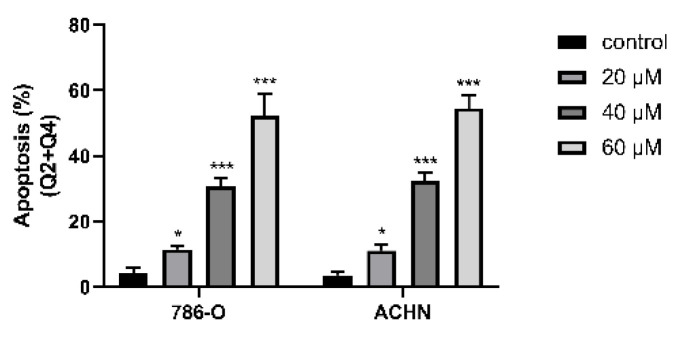
Correct image of Figure S1B.

