# Supplementary material for: Cyclovirobuxine inhibits the progression of clear cell renal cell carcinoma by suppressing the IGFBP3-AKT/STAT3/MAPK-Snail signalling pathway
Source: Int J Biol Sci. 2021 Aug 13;17(13):3522–37. doi: 10.7150/ijbs.62114 (PMC8416721; doi:10.7150/ijbs.62114)
Supplement: Supplementary file 1 — Supplementary figures and tables. [file ijbsv17p3522s1.pdf]

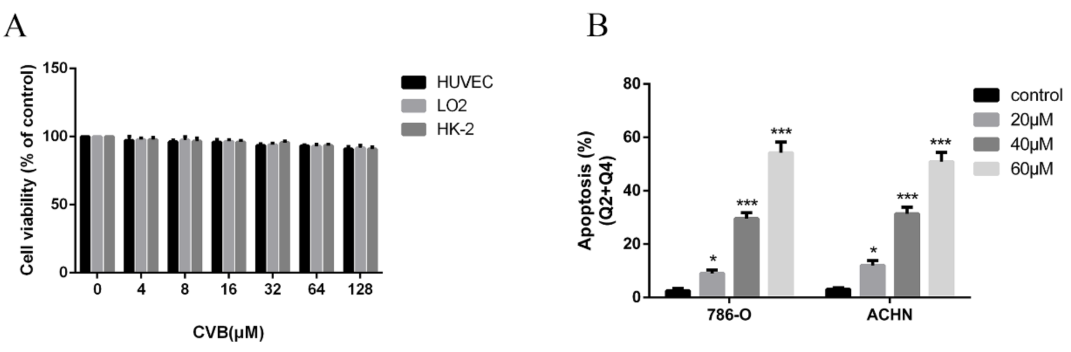

Fig S1. (A) Effect of various doses of CVB (0-128 μM, 48 h) on the viability of normal human cells HUVEC, L0-2 and HK-2 cells. (B) Apoptosis of 786-O and ACHN cells after treatment with CVB.

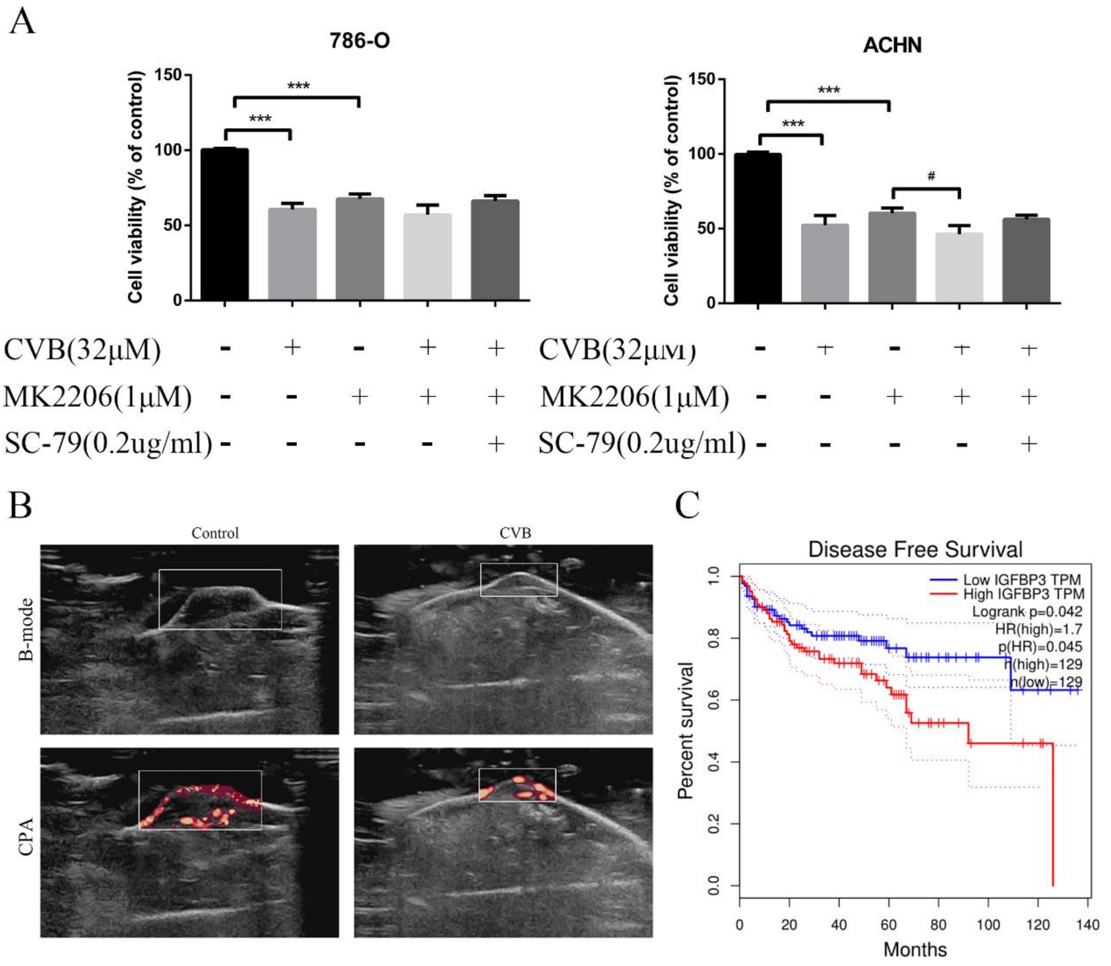

Fig S2. (A) Effect of CVB, MK2206 and SC-79 on the viability of 786-O and ACHN cells. (B) Ultrasonography evaluation subcutaneous tumors included B-mode and CPA. (C) The DFS of RCC patients in IGFBP3-low and IGFBP3-high groups (GEPIA).

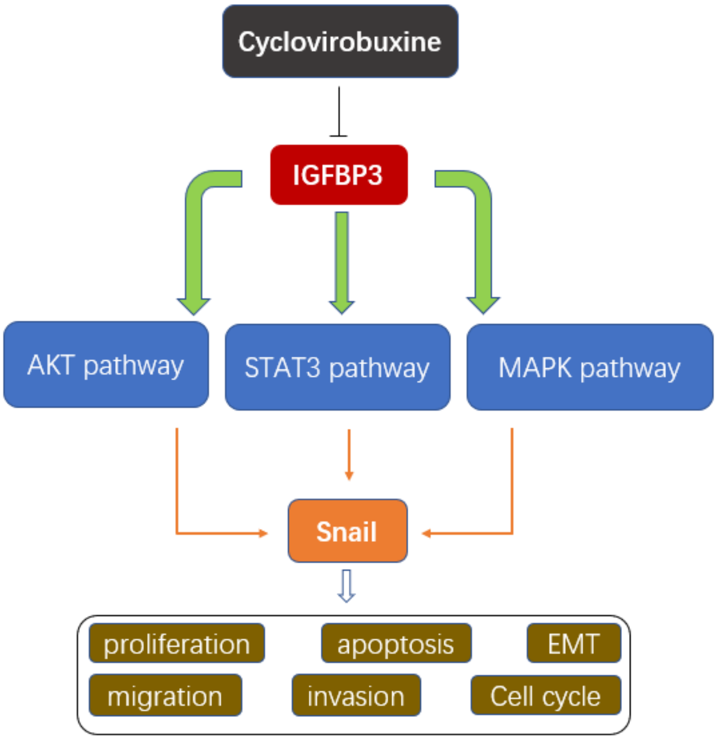

Fig S3. Diagram illustrating the signaling pathway involved in the CVB-induced inhibition of ccRCC.

Table S1. The siRNA sequences for the IGFBP3 gene.

siRNA	Sense (5'-3')
Si-NC (random control sequence)	UGACCUCAACUACAUGGUUTT
si-IGFBP3-1	CUCCAUUCAAAGAUAAUCATT
si-IGFBP3-2	CUGCCGUAGAGAAAUGGAATT
si-IGFBP3-3	GGUGUACACAUUCCCAACUTT
